# Threonine 32 (Thr32) of FoxO3 is critical for TGF-β-induced apoptosis via Bim in hepatocarcinoma cells

**DOI:** 10.1007/s13238-014-0121-5

**Published:** 2014-12-11

**Authors:** Xiangxuan Zhao, Yong Liu, Lei Du, Leya He, Biyun Ni, Junbo Hu, Dahai Zhu, Quan Chen

**Affiliations:** 1 The Joint Laboratory of Apoptosis and Cancer Biology, The State Key Laboratory of Biomembrane and Membrane Biotechnology, Institute of Zoology, Chinese Academy of Sciences, Beijing, 100101 China; 2 University of Chinese Academy of Sciences, Beijing, 100049 China; 3 Department of Radiology, Shengjing Hospital of China Medical University, Shenyang, 110004 China; 4 Cancer Research Center, Tongji Hospital, Tongji Medical College, Huazhong University of Science and Technology, Wuhan, 430032 China; 5 Institute of Basic Medical Sciences of Chinese Academy of Medical Sciences and School of Basic Medicine of Peking Union Medical College, Beijing, 100005 China; 6 College of Life Science, Nankai University, Tianjin, 300071 China

**Keywords:** apoptosis, TGF-β, FoxO3, casein kinase I-ε, hepatocarcinoma

## Abstract

Transforming growth factor-β (TGF-β) exerts apoptotic effects on various types of malignant cells, including liver cancer cells. However, the precise mechanisms by which TGF-β induces apoptosis remain poorly known. In the present study, we have showed that threonine 32 (Thr32) residue of FoxO3 is critical for TGF-β to induce apoptosis via Bim in hepatocarcinoma Hep3B cells. Our data demonstrated that TGF-β induced FoxO3 activation through specific de-phosphorylation at Thr32. TGF-β-activated FoxO3 cooperated with Smad2/3 to mediate Bim up-regulation and apoptosis. FoxO3 (de)phosphorylation at Thr32 was regulated by casein kinase I-ε (CKI-ε). CKI inhibition by small molecule D4476 could abrogate TGF-β-induced FoxO/Smad activation, reverse Bim up-regulation, and block the sequential apoptosis. More importantly, the deregulated levels of CKI-ε and p32FoxO3 were found in human malignant liver tissues. Taken together, our findings suggest that there might be a CKI-FoxO/Smad-Bim engine in which Thr32 of FoxO3 is pivotal for TGF-β-induced apoptosis, making it a potential therapeutic target for liver cancer treatment.

## INTRODUCTION

TGF-β mainly signals through activating a heteromeric receptor complex consisting of type I (TGF-RI) and type II (TGF-RII) serine/threonine kinase on the cell membrane (Massague and Weis-Garcia, 1996). The activated TGF-β receptors phosphorylate downstream adaptor proteins such as Smad2 and Smad3. Receptor-activated Smads are associated with a common Smad4 and translocate to the nucleus to modulate TGF-β target genes (Derynck and Zhang, 2003; Enroth et al., 2014; Engel et al., 1998; Massague et al., 2005). Increasing evidence indicated that Smad proteins cooperate with a variety of transcription factors, including AP-1, TFE3 and FoxO to activate gene transcription or repress gene expression in association with oncoproteins such as Evi-1, E1A, Ski, SnoN, Tid1 and Akt (Hua et al., 1998; Remy et al., 2004; Runyan et al., 2012; Torregroza and Evans, 2006; Vignais, 2000; Yamamura et al., 2000; Seoane et al., 2002). TGF-β can induce apoptosis in malignant cells through up-regulating of pro-apoptotic proteins such as Bim and Bmf, or down-regulating anti-apoptotic proteins such as Bcl-xL (Nass et al., 1996; Ramjaun et al., 2007).

The FoxO transcription factor family, including FoxO1, FoxO3 and FoxO4 is reported to act as potent transcription activators and tumor suppressors. Specifically, FoxO3 is phosphorylated by a couple of protein kinases such as PKB/Akt and CKI (Conery et al., 2004; Waddell et al., 2004). Once phosphorylated, FoxO3 is sequestrated in the cytoplasm and its ability to activate transcription of target genes is inhibited. It has been reported that TGF-β induces FoxO3 to actively engage with Smads to result in cell cycle arrest by up-regulating p27 (Park et al., 2013; Kato et al., 2006). Studies also showed that TGF-β enhances FoxO3 phosphorylation and down-regulates Bim expression to inhibit apoptosis in mesangial cells (Naka et al., 2010). However, the roles of FoxO3 in TGF-β-induced apoptosis in liver cancer cells have yet to be fully elucidated.

Casein kinase I (CKI) family proteins consisting of seven isoforms (α, β, γ1–3, δ and ε) can phosphorylate p53 or β-catenin to regulate their activity; Of note, CKI-ε is regarded as a constitutively active kinase and its activity is regulated by (auto) phosphorylation status (Fish et al., 1995; Tuazon and Traugh, 1991; Knippschild et al., 2005; Graves et al., 1993). Previous studies reported that CKI-ε can enhance TGF-β-induced Smad-mediated gene transcription (Renard et al., 2008; Miyazono, 2000). Currently, the mechanism by which CKI-ε regulates FoxO3 activity to affect TGF-β-induced apoptosis remains unclear.

The present study sought to study the roles of FoxO3 in TGF-β-induced apoptosis using *in vitro* cell models. We proved that TGF-β triggers apoptosis via Bim elevation in Hep3B cells. TGF-β activated FoxO3 by dephosphorylation at Thr32 and the activated FoxO3 functionally cooperated with Smad2/3 to mediate Bim up-regulation. CKI-ε regulated FoxO3 activity by Thr32 phosphorylation site and affected TGF-β-induced Bim up-regulation and apoptosis. Deregulated expression of CKI-ε and p32FoxO3 was observed in malignant liver tissues. Our findings suggest that a CKI-ε-FoxO3/Smad-Bim engine could be considered as a potential target to treat liver cancer.

## RESULTS

### TGF-β induces Bim-dependent apoptosis in Hep3B cells

**Figure 1:**
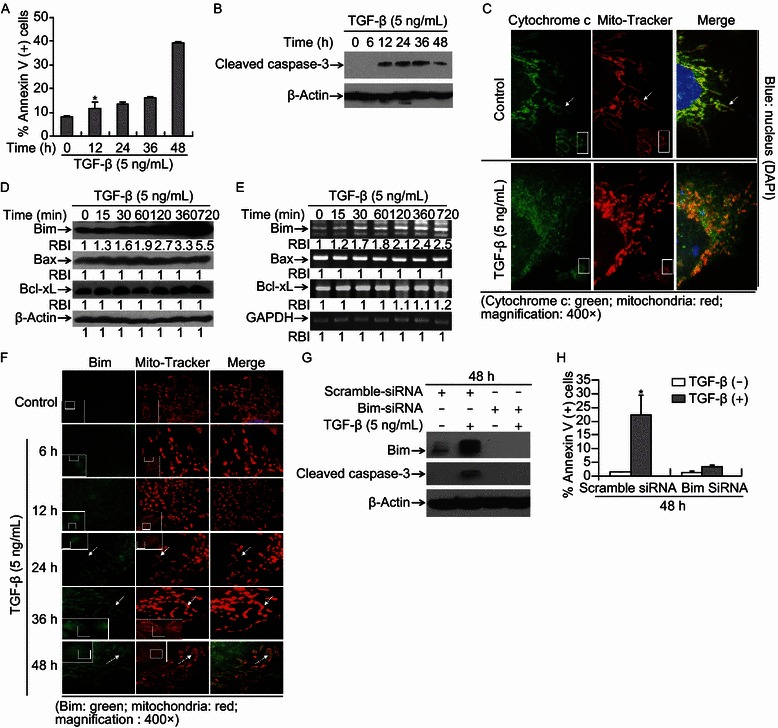
**TGF-β induces Bim dependent apoptosis in Hep3B cells**. (A) TGF-β-induced apoptosis in Hep3B cells. Cells treated with TGF-β (5 ng/mL) for up to 48 h were harvested and processed for apoptotic assay by using the Annexin V-PI double staining as described in ‘METHODS AND MATERIALS’. Statistical analysis was carried out to assess the ratio of apoptosis. Representative data were shown and every experiment was repeated three times (*P < 0.05). (B) Detection of apoptosis by caspase-3 activation. Cells treated as in Fig. 1A were harvested and cell lysates were prepared for Western blotting analysis to detect levels of cleaved caspase-3 with specific antibody recognizing cleaved caspase-3. β-Actin protein levels were assessed as loading controls for equal total protein amounts. Representative immuno-bands were shown and every experiment was repeated three times. (C) Cytochrome c release from mitochondria. Cells cultured on glass cover slips were treated with TGF-β (5 ng/mL) for 48 h or not (Control). Fluorescence immunostaining was performed to detect cytochrome c with anti-cytochrome c primary mouse antibody. Bound cytochrome c was labeled with FITC conjugated goat anti-mouse secondary antibody (green). Mitochondria were stained with Mito-Tracker (Red). (D) TGF-β-induced Bim up-regulation at protein level. Cells incubated in the absence or presence of TGF-β for up to 720 min were harvested and cell lysates were prepared for Western blotting to detect levels of Bim, Bax and Bcl-xL with antibodies specifically recognizing Bim, Bax and Bcl-xL respectively. β-Actin protein levels were assessed as loading controls for equal total protein amounts. Relative band intensities (RBIs) were analyzed by the Image J software. (E) TGF-β-induced Bim up-regulation at mRNA level. Cells were treated as in D and semi-quantitative RT-PCR was used to detect levels of Bim, Bax and Bcl-xL mRNA. GAPDH mRNA were assessed and set up as equal loading control. Relative band intensities (RBIs) were analyzed by the Image J software. (F) Bim up-regulation and translocation. Cells cultured on cover slips were treated for up to 48 h or not. Immunofluorescence staining was performed as described in ‘MATERIALS AND METHODS’. Bim was recognized with anti-Bim rabbit pAb and bound Bim primary antibody was labeled with FITC conjugated goat anti-rabbit IgG (Green). Mitochondria were stained with Mito-Tracker (Red). (G) TGF-β-induced Bim-dependent apoptosis. Cells cultured in six-well plate were transfected with synthesized scramble control siRNA and Bim specific siRNA, and 48 h post-transfection, cells were treated with TGF-β (5 ng/mL) for 48 h or not. Bim expression and apoptotic effects based on caspase-3 cleavage activation were determined through Western blotting. (H) Cells cultured in six-well plate were transfected with synthesized scramble control siRNA and Bim specific siRNA, and 48 h post-transfection, cells were treated with TGF-β (5 ng/mL) for 48 h or not. Apoptotic ratio was determined by counting cells with apoptotic nuclei as described in ‘MATERIALS AND METHODS’. Data represent the mean values of three independent experiments (*P < 0.05)

To evaluate the apoptotic effects of TGF-β, Hep3B cells were treated with TGF-β. Apoptosis was determined by FACS analysis based on Annexin V-PI double staining, caspase-3 cleavage activation and cytochrome c release from mitochondria. We observed significant apoptosis in TGF-β-treated cells (Fig. 1A–C). Regarding Bcl-2 family proteins are essential regulators of cytochrome c release from mitochondria (Green and Reed, 1998), next we analyzed the expression of Bcl-2 family proteins in Hep3B cells treated with TGF-β. We found that Bim was significantly up-regulated at both protein and mRNA levels, while Bax and Bcl-xL were not apparently affected (Fig. 1D and 1E). To further verify the roles of Bim in Hep3B cells treated with TGF-β, immunofluorescence staining assays were performed. Our data showed that Bim was elevated and translocated to mitochondria in cells treated with TGF-β (Fig. 1F), suggesting Bim may play key roles in cytochrome c release from mitochondria. To validate whether TGF-β-induced apoptosis is Bim dependent, we used the siRNA system to suppress the expression of Bim. Western blotting results indicated that Bim expression was effectively knocked down in Bim specific siRNA-transfected cells (Fig. 1G). Apoptosis assays revealed that Bim knock-down effectively protected cells against TGF-β-induced apoptosis (Fig. 1G and 1H). These results suggest that TGF-β-induced apoptosis is Bim dependent in Hep3B cells.

### FoxO3 and Smad2/3 are activated and cooperate to mediate Bim up-regulation

**Figure 2:**
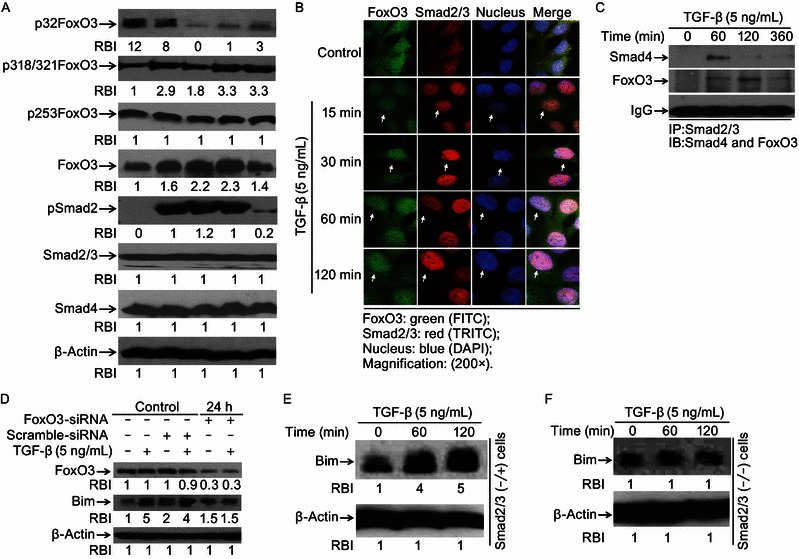
**FoxO3 and Smad2/3 are activated and cooperate to mediate Bim up-regulation**. (A) TGF-β induces FoxO3 dephosphorylation at Thr32. Cells treated with TGF-β (5 ng/mL) for indicated times were harvested and cell lysates were prepared for Western blotting to examine protein levels of p32FoxO3, p253FoxO3, p318/321FoxO3, FoxO3, Smad2/3, pSmad2 and Smad4 with specific antibodies respectively. Levels of equal protein loading were determined by β-Actin. Relative band intensities (RBIs) were analyzed by the Image J software. (B) Smad2/3 and FoxO3 co-translocation to nucleus. Hep3B cells treated with TGF-β (5 ng/mL) for indicated times were fixed with paraformaldehyde and immunostained with anti-Smad2/3 or anti-FoxO3 primary antibodies. Bound Smad2/3 antibody was recognized with Cy3-conjugated donkey anti-mouse IgG (Red) and FoxO3 antibody was recognized with FITC-conjugated goat anti-rabbit IgG (Green). (C) TGF-β-induced FoxO3-Smad2/3 complex formation. Cells treated for 60, 120 and 360 min or not were harvested and cell lysates were prepared for immunoprecipitation with anti-Smad2/3 followed by immunoblotting with anti-Smad4 mAb or anti-FoxO3 pAb. Equal protein amounts loading were determined by IgG. (D) FoxO3 knockdown blocks TGF-β-induced Bim up-regulation. Cells were transfected with FoxO3 specific interfering oligonucleotides (FoxO3-siRNA) or non-specific oligonucleotides scramble-siRNA. After transfection for 24 h, cells treated with TGF-β were harvested and cell lysates were prepared for Western blotting to detect levels of FoxO3 and Bim with specific antibodies recognizing FoxO3 and Bim. Relative band intensities (RBIs) were analyzed by the Image J software. (E) and (F) Smads knockout abolishes TGF-β-induced Bim up-regulation. Smad2/3^−/−^and Smad2/3^−/+^ MEF cells treated with TGF-β (5 ng/mL) were harvested and cell lysates were prepared for Western blotting to detect Bim with Bim pAb. Levels of equal protein loading were determined by β-Actin. Relative band intensities (RBIs) were analyzed by the Image J software. Representative bands were shown. Each experiment was conducted in triplicate and repeated twice independently

FoxO3 is a key transcription factor to regulate Bim gene expression (Hagenbuchner et al., 2012). In order to test the function of FoxO3 in TGF-β-induced apoptosis, cells were treated with TGF-β and Western blotting was performed. We found that FoxO3 was dephosphorylated at threonine 32 (Thr32) residue after treatment with TGF-β for 30 min, but the other three serine 253, 318, or 321 residues were not (Fig. 2A). FoxO3 was increased after treatment with TGF-β for 120 min, which may be caused by dephosphroylation of p32FoxO3 (Fig. 2A). TGF-β triggered dramatic Smad2/3 phosphorylation within 15 min and the protein levels of both Smad2/3 and smad4 were not apparently affected (Fig. 2A). It is well documented that activated FoxO3 could move into nucleus to regulate target gene expression. Here we observed that TGF-β-induced Smad2/3 phosphorylation activation mirrored FoxO3 Thr32 dephosphorylation activation (Fig. 2A). To assess whether FoxO3 and Smad2/3 could be activated simultaneously and cooperate to regulate target gene transcription, double immunostaining assays were performed. Our data demonstrated that TGF-β treatment induced FoxO3 translocation into the nucleus in a similar time course with Smad2/3 (Fig. 2B). Co-immunoprecipitation (co-IP) experiments further confirmed that TGF-β was able to stimulate Smad2/3 and FoxO3 to form a complex (Fig. 2C). Additionally, our CHIP assays and oligonucleotide pull-down assays ascertained that Smad-FoxO3 complex could bind to *bim* promoter (data not shown). To verify the functional involvement of Smad2/3 and FoxO3 to TGF-β-induced Bim elevation, we used the siRNA system to suppress the expression of FoxO3 and found that FoxO3-siRNA effectively abrogated TGF-β-induced Bim up-regulation (Fig. 2D). To test the roles of Smad2/3, Smad knockout (Smad2/3^−/−^) and Smad heterozygous (Smad2/3^−/+^) MEF cells were used to evaluate TGF-β-induced Bim increase. We observed that Bim was significantly increased in Smad2/3^−/+^ MEF cells (Fig. 2E), but not in Smad2/3^−/−^ MEF cells (Fig. 2F). These data suggest that both FoxO3 and Smad2/3 are activated and cooperate to regulate TGF-β-induced Bim up-regulation.

### PI3K-Akt pathway is not involved in FoxO3 activation induced by TGF-β

**Figure 3:**
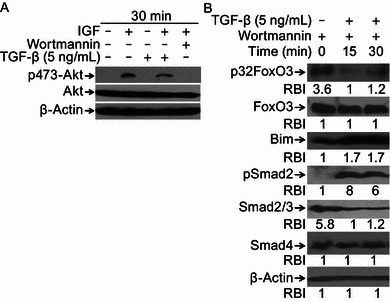
**PI3K-Akt pathway is not involved in TGF-β-induced FoxO3 activation**. (A) TGF-β treatment does not induce Akt phosphorylation activation. Hep3B cells were treated with IGF-1, TGF-β, IGF-1 and TGF-β or IGF-1 and Wortmannin for 30 min. Cells were harvested and cell lysates were prepared for Western blotting to detect levels of p473-Akt and Akt with antibodies specifically recognizing p473-Akt and Akt. β-Actin was assessed and set up as equal protein loading control. (B) Inhibition of PI3K-Akt does not block TGF-β-induced FoxO3 activation and Bim up-regulation. Hep3B cells pretreated with Wortmannin for 30 min were stimulated with TGF-β (5 ng/mL) or not for 0, 15 and 30 min. Cell lysates were prepared and subjected to Western blotting to detect levels of p32FoxO3, Bim, FoxO3, pSmad2, Smad2/3, Smad4 with specific antibodies, respectively. β-Actin levels were assessed and set up as equal protein loading control. Relative band intensities (RBIs) were analyzed by the Image J software. Representative bands were shown. Each experiment was conducted in triplicate and repeated twice independently

Depending on the cell types, TGF-β inhibits or activates Akt through changing its phosphorylation status. Previous studies has shown that Akt is involved in phosphorylation of FoxO3 at Thr32 (Higaki and Shimokado, 1999; Valderrama-Carvajal et al., 2002). In our study, to verify whether FoxO3 Thr32 dephosphorylation was mediated by Akt, cells were treated or not with insulin-like growth factor 1 (IGF-1) (positive control), TGF-β, IGF-1 plus TGF-β, or IGF-1 plus Wortmannin (negative control). Our results indicated that TGF-β did not induce Akt phosphorylation activation in Hep3B cells (Fig. 3A). To further confirm PI3K/Akt pathway was not involved in TGF-β-induced FoxO3 activation at Thr32, cells were treated with TGF-β in the presence of Wortmannin or not. We found that TGF-β-induced dephosphorylation of FoxO3 at Thr32 and Bim up-regulation was not apparently affected (Fig. 3B). Our results also indicated that Smad2 was phosphorylated and Smad4 had no change. Notably, we found that TGF-β and Wortmannin combination treatment led to Smad2/3 decrease, of which the underlying mechanism needs further investigation. Taken together, these data suggest that TGF-β-induced FoxO3 activation through dephosphorylation at Thr32 is not PI3K-Akt dependent.

### CKI-ε regulates TGF-β-induced Bim up-regulation through FoxO3 in Hep3B cells

**Figure 4:**
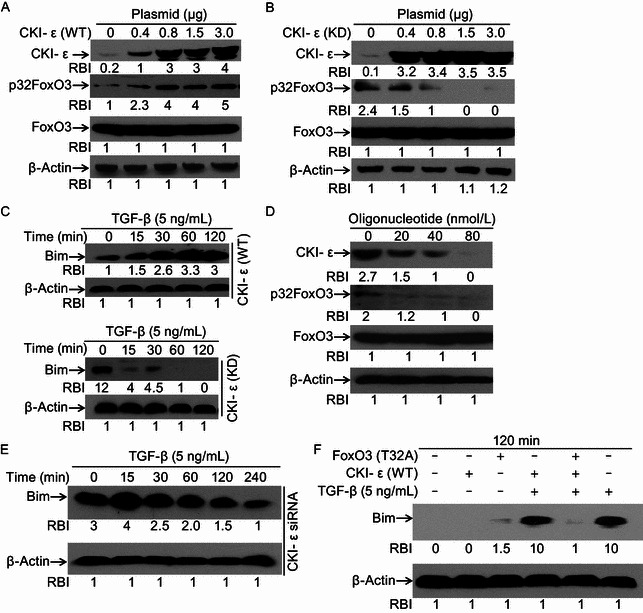
**CKI-ε regulates TGF-β-induced Bim up-regulation through FoxO3**. (A) Over-expression of CKI-ε (WT) induces p32FoxO3 increase. Hep3B cells were transiently transfected with different amounts of wild type CKI-ε (WT) plasmids. After transfection for 48 h, cell lysates were prepared for Western blotting to detect levels of CKI-ε and p32FoxO3. Equal protein loading was determined by β-Actin. Relative band intensities (RBIs) were analyzed by the Image J software. (B) Mutation of CKI-ε impairs its ability to phosphorylate FoxO3 at Thr32. Hep3B cells were transiently transfected with dominant negative mutant CKI-ε (KD) (K to R). After transfection for 48 h, cells were harvested and lysates were prepared for Western blotting. CKI-ε and Bim expression were evaluated with specific antibodies recognizing CKI-ε and Bim. β-Actin was assessed and set up as equal protein loading control. Relative band intensities (RBIs) were analyzed by the Image J software. (C) Effects of CKI-ε over-expression on TGF-β-induced Bim. Hep3B cells transiently transfected with either wild type CKI-ε (WT) (upper panel) or dominant negative CKI-ε (KD) (lower panel) plasmids were treated with TGF-β for up to 120 min or not. Cells were harvested and lysates were prepared for Western blotting to assess Bim expression. Equal protein loading was determined by β-Actin. Relative band intensities (RBIs) were analyzed by the Image J software. (D) CKI-ε knockdown decreases p32FoxO3 levels. Hep3B cells were transfected with CKI-ε specific siRNA or not. After transfection for 48 h, cells were harvested and cell lysates were prepared for Western blotting to assess levels of CKI-ε and p32FoxO3 with antibodies specifically recognizing CKI-ε and p32FoxO3. β-Actin was set up as equal protein loading control. Relative band intensities (RBIs) were analyzed by the Image J software. (E) CKI-ε knockdown reverses TGF-β-induced Bim up-regulation. Hep3B cells were transfected with CKI-ε siRNA or not. After transfection for 48 h, cells were harvested and cell lysates were prepared for Western blotting to assess level of Bim. Equal protein loading was determined by β-Actin. Relative band intensities (RBIs) were analyzed by the Image J software. (F) FoxO3 mutation at Thr32 impairs TGF-β-induced Bim up-regulation. Hep3B cells, Hep3B cells transiently transfected with CKI-ε (WT) plasmids, Hep3B cells transiently transfected with FoxO3 (T to A) plasmids, or Hep3B cells transiently transfected with both CKI-ε (WT) and FoxO3 (T to A) plasmids were treated with TGF-β or not for 120 min. Bim expression was monitored by Western blotting with anti-Bim pAb. β-Actin was assessed and set up as equal protein loading control. Relative band intensities (RBIs) were analyzed by the Image J software. Representative bands were shown. Each experiment was conducted in triplicate and repeated twice independently

CKI-ε plays a ligand-dependent regulatory role in the TGF-β signaling pathway. CKI-ε can repress basal activity of TGF-β-targeted molecules, while enhance TGF-β-induced Smad-mediated gene transcription (Higaki and Shimokado, 1999; Valderrama-Carvajal et al., 2002). In this study, we tested whether CKI-ε affects FoxO3 phosphorylation status. Western blotting results indicated that over-expression of wild type CKI-ε (WT) effectively resulted in p32FoxO3 increase (Fig. 4A), whereas over-expression of dominant-negative mutant CKI-ε (KD) decreased p32FoxO3 level (Fig. 4B). To assess the effects of CKI-ε-mediated FoxO3 phosphorylation at Thr32 on TGF-β-induced Bim up-regulation, Hep3B cells harboring CKI-ε (WT) or CKI-ε (KD) plasmids were treated with TGF-β or not. We found that TGF-β increased Bim expression in cells expressing wild type CKI-ε (Fig. 4C, upper panel). TGF-β-induced Bim up-regulation was reversed in cells containing CKI-ε (KD) (Fig. 4C, lower panel). We next checked the roles of endogenous CKI-ε in FoxO3 Thr32 phosphorylation activation and Bim expression. Using siRNA to knock down CKI-ε expression, Western blotting results showed that CKI-ε knockdown effectively decreased p32FoxO3 level (Fig. 4D). More interestingly, we found that TGF-β-induced Bim up-regulation was reverted in CKI-ε knockdown cells (Fig. 4E), which is consistent with the results from TGF-β-treated CKI-ε (KD) containing cells. To further verify that Threonine 32 (Thr32) residue of FoxO3 is critical for Bim expression, FoxO3 plasmid containing a point mutation (Thr32 to Ala32: T to A) was introduced to cells. After 48 h transfection, cells treated with TGF-β or not. We found that over-expression of this mutant FoxO3 ablated TGF-β-induced Bim increase (Fig. 4F). Taken together, these data suggest that CKI-ε plays pivotal roles in regulating FoxO3 phosphorylation at Thr32.

### CKI inhibition blocks TGF-β-induced Bim increase and apoptosis

**Figure 5:**
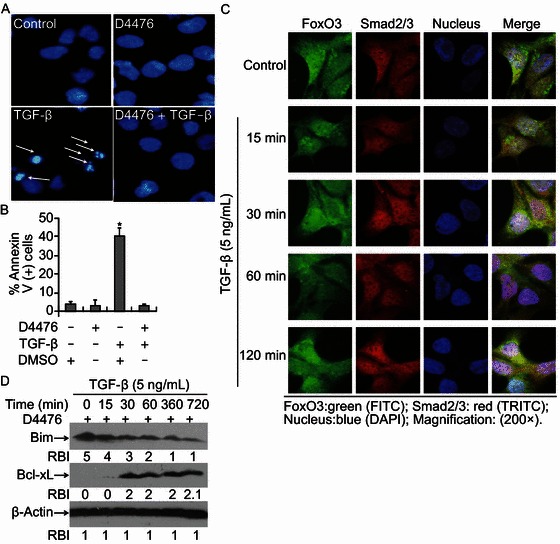
**CKIinhibition blocks TGF-β-induced apoptosis in Hep3B cells**. (A) CKI-ε inhibition blocks TGF-β-induced apoptosis. Cells were treated with CKI inhibitor D4476 (10 μmol/L), TGF-β (5 ng/mL) or D4476 plus TGF-β for 48 h. Apoptosis was assessed by staining with Hoechst 33342 for nucleus condensation as described in ‘MATERIALS AND METHODS’. A representative field of cells with the indicated treatments has been shown. Typically apoptotic cells with apoptotic nuclei were marked with white arrows. (B) Cells were treated with various conditions as indicated. Apoptosis was assessed by FACS analysis based on Annexin V-PI double staining. Statistical analysis was performed to assess the ratio of apoptosis. Data represent the mean values of three independent experiments (*P < 0.05). (C) D4476 abolishes TGF-β-stimulated Smad2/3 and FoxO3 translocation. Hep3B cells grown on cover slips were pretreated with D4476 for 30 min and continuously incubated with TGF-β (5 ng/mL) or not. Immunofluorescence double staining was performed to evaluate the presence of Smad2/3 and FoxO3 with antibodies specifically recognizing Smad2/3 and FoxO3. The bound Smad2/3 primary antibody was visualized with Cy3-conjugated donkey anti-mouse IgG (Red) and FoxO3 was with FITC-conjugated goat anti-rabbit IgG (Green). Nuclei were stained with specific dye DAPI (blue). (D) D4476 blocks TGF-β-induced Bim up-regulation. Cells were pretreated with D4476 for 30 min followed by TGF-β (5 ng/mL) incubation for various times ranging from 0 to 720 min. Cells were harvested and cell lysates were prepared for Western blotting to detect levels of Bim and Bcl-xL with specific antibodies recognizing Bim and Bcl-xL. Equal protein loading was determined by β-Actin. Relative band intensities (RBIs) were analyzed by the Image J software. Representative bands were shown. Each experiment was conducted in triplicate and repeated twice independently

To further evaluate the function of CKI-ε in TGF-β-induced apoptosis, cells were pretreated with CKI inhibitor D4476 followed by TGF-β incubation. Apoptosis was measured by nuclear staining and Annexin V-PI staining. We found that CKI inhibition by D4476 effectively protected cell against TGF-β-induced apoptosis (Fig. 5A and 5B). Immunofluoresence staining assays demonstrated that TGF-β-induced translocation of both Smad2/3 and FoxO3 was completely blocked by D4476 (Fig. 5C). Western blotting results indicated that TGF-β-induced Bim up-regulation was reversed by this inhibitor (Fig. 5D). These data further suggest that CKI-ε is correlated with TGF-β-induced FoxO3 activation and Bim up-regulation.

### Deregulation of CKI-ε and p32FoxO3 in hepatocarcinoma

**Figure 6:**
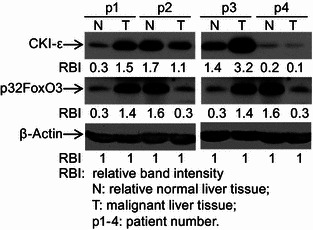
**Deregulation of CKI-ε and p32FoxO3 in hepatocarcinoma**. Paired adjacent normal liver tissues (N) and malignant liver tissues (T) from 4 patients suffered hepatocarcinoma were collected and processed for Western blotting to detect levels of CKI-ε and p32FoxO3 with specific antibodies recognizing CKI-ε and p32FoxO3. Equal protein loading was determined by β-Actin. Relative band intensities (RBIs) were analyzed by the Image J software. N: samples from adjacent liver tissues; T: samples from malignant liver tissues; p1–4: patient number. Representative bands were shown. Each experiment was conducted in triplicate and repeated twice independently

It has been reported that cancer cells may have deregulated apoptosis signaling pathway (Huynh et al., 2003; Philips and McFadden, 2004; Akagi et al., 1996). To assess whether malignant liver cells have aberrant CKI-ε and p32FoxO3 expression that reduces the sensitivity of cancer cells to TGF-β-induced apoptosis. Paired malignant liver tissues and adjacent non-cancer tissues from the same patients were collected. CKI-ε and p32FoxO3 expression was analyzed by Western blotting. Matched comparisons with adjacent normal liver tissues showed that there was consistent down-regulation of CKI-ε and p32FoxO3 protein expression in 2 out of 4 randomly selected malignant liver tissues (Fig. 6). These data suggest that the CKI-ε activity and phosphorylation of FoxO3 are deregulated in human liver cancer cells.

## DISCUSSION

In this study, we have addressed the mechanism of TGF-β-induced Bim-dependent apoptosis in malignant liver cells. We found that TGF-β induced FoxO3 activation by dephosphorylation at Thr32. Activated FoxO3 cooperated with Smad2/3 to mediate Bim up-regulation. CKI-ε regulated TGF-β-induced Bim elevation and apoptosis by affecting FoxO3 phosphorylation status at Thr32. CKI inhibition effectively blocked TGF-β-induced apoptosis. More importantly, we observed deregulation of CKI-ε and p32FoxO3 in liver cancer tissues. Our results delineate a CKI-FoxO/Smad-Bim engine in which Thr32 of FoxO3 is critical for TGF-β-induced apoptosis.

Under TGF-β stimulation, FoxO3 can bind to Runx-1 and cooperate to up-regulate Bim expression (Wildey and Howe, 2009). However, which phosphorylation site(s) of FoxO3 play(s) key roles in TGF-β-induced FoxO3 activation or whether FoxO3 can function as a cofactor with smad2/3 to regulate TGF-β-induced apoptosis in liver cancer cells has yet to be fully tested. In this study, we demonstrate that TGF-β induced FoxO3 activation through specific dephosphorylation at Thr32 residue. Interestingly, the dephosphorylation activation pattern of FoxO3 was matched (mirrored) perfectly with phosphorylation activation of Smad2/3, suggesting the possibility of FoxO3 and Smad2/3 co-operation. We also observed that FoxO3 and Smad translocated to nuclear from cytoplasm simultaneously. Moreover, the following co-IP assay further confirmed that FoxO3 could be a co-factor of Smad2/3. Loss-of-function assay using siRNA-mediated FoxO3 knockdown Hep3B cells or Smad2/3 knockout MEF cells revealed that both FoxO3 and Smad2/3 were essential for TGF-β-induced Bim-dependent apoptosis. Therefore, these results provided profound evidence to validate the notion that TGF-β induced FoxO3 Thr32 dephosphorylation and activated FoxO3 could cooperate with Smad2/3 to medicate Bim up-regulation.

FoxO3 dephosphorylation activation could be achieved either by phosphatases or kinases (Vogt et al., 2005). It has been reported that TGF-β could activate PP2A, which might lead to dephosphorylation of FoxO3 (Ni et al., 2007; Tremblay and Giguere, 2008; Yan et al., 2008). However, our present data indicated that inhibition of PP2A by a specific inhibitor Okadaic Acid did not prevent TGF-β-induced FoxO3 activation (data not shown). Previous reports also showed that Akt can phosphorylate FoxO3 at Thr32 to inhibit its activity (Brunet et al., 1999; Biggs et al., 2001; Kops and Burgering, 1999). In our study, we found that TGF-β did not induce Akt activation and Wortmannin could not affect TGF-β-stimulated FoxO3 dephosphorylation at Thr32, suggesting that PI3K/Akt was not involved in TGF-β-induced activation of FoxO3. Therefore, it would be interesting to explore the possible kinase(s) that may modulate TGF-β-induced FoxO3 activation. Our study indicated that over-expression of wild type CKI-ε (WT) significantly induced p32FoxO3 increase, while the dominant negative CKI-ε (KD) over-expression caused 32FoxO3 decrease, suggesting that CKI-ε is a potential protein kinase for maintaining FoxO3 Thr32 phosphorylation. More importantly, we observed that ectopic over-expression of CKI-ε (KD) result in Bim decrease in TGF-β-treated cells. Thus, we hypothesized that CKI-ε may be responsible for controlling the direction of the CKI-FoxO/Smad-Bim engine. Indeed, TGF-β-induced Bim up-regulation was also reversed in CKI-ε knockdown cells, suggesting CKI-ε is correlated with TGF-β-induced FoxO/Smad-mediated Bim up-regulation. Additionally, ablation of Bim up-regulation by expressing mutant FoxO3 (T32A) plasmid in cells harboring CKI-ε (WT) further confirmed that both CKI-ε and p32FoxO3 are crucial.

Based on the above observation that CKI-ε acts as a protein kinase to regulate FoxO3 biological activity through Thr32 residue and determines Bim expression profile, we speculate that inhibition of CKI-ε may inhibit TGF-β-induced Bim increase and apoptosis. As expected, we found that TGF-β-induced apoptosis was completely blocked by CKI inhibitor D4476. Further studies revealed that D4476 incubation not only impaired FoxO3 and Smad2/3 collaborating activation, but altered TGF-β-induced Bim expression profile from increase to decrease. This is consistent with our findings that CKI-ε (KD) over-expression or CKI-ε knockdown revert TGF-β-induced Bim expression. Taken together, these data make it clear that CKI-ε is able to regulate TGF-β-induced Bim expression by affecting FoxO3 phosphorylation status and support the model in which TGF-β stimulates apoptosis by trigger the CKI-FoxO/Smad-Bim engine.

In an attempt to bring our studies close to clinical analysis, we then demonstrate that, in comparison with adjacent normal liver tissues, 2 out of 4 malignant liver tissues have lower CKI-ε and p32FoxO3 expression. Since the tumor suppressive actions of the FoxO3 are well documented (Renault et al., 2011; Qi et al., 2011a, b; Karube et al., 2011), our findings imply that massive inactivation of FoxO3 by Thr32 phosphorylation may either initiate a progressive cancer-prone condition or have a pro-metastatic role to promote tumor progression in liver cancer.

In summary, our data reveal a novel CKI-FoxO/Smad-Bim engine for TGF-β-induced apoptosis and its deregulation may be related to cancer development. Identification of this engine may provide a potential new therapeutic target in the treatment of liver cancer, although it remains to be defined what happens to CKI-ε upon TGF-β stimulation.

## Materials and methods

### Materials and cell culture

Primary antibodies: to Bim rabbit pAb (BOD) and β-Actin mouse mAb (Sigma-Aldrich); to cytochrome c mouse mAb (BD Pharmingen); to Smad2/3 mouse mAb (BD Transduction laboratories); to p-Akt (473) rabbit pAb, caspase-3 rabbit pAb, p-Smad2 mAb, p-FoxO3 (Thr32/Ser318/321/Thr253) rabbit pAb and Akt rabbit pAb (Cell Signaling Technology); to Smad4 mouse mAb (B-8) and FoxO3 rabbit pAb (Santa Cruz Biotechnology). Second antibodies: FITC conjugated goat anti-rabbit IgG (Santa Cruz Biotechnology); Cy3 conjugated donkey anti-mouse IgG (Jackson ImmunoResearch Lab, INC.); Wortmannin (Upstate); IGF-1 (Insulin-like growth factor 1, IGF-1) and human TGF-β was obtained from R&D systems; Protein G Plus-Sepharose (Santa Cruz Biotechnology, INC); PI (Propidium Iodide, Sigma-Aldrich); DAPI (4,6-Diamidino-2-phenyindole, DAPI) and Hoechst 33342 were from Sigma–Aldrich, Inc.; Superscript-TM II reverse Transcriptase kit (Invitrogen); Hep3B cells were cultured in minimum essential medium (MEM) supplemented with 10% fetal bovine serum, 100 units/mL penicillin, and 100 mg/mL streptomycin at 37°C and 5% CO_2_. MEF cells, Smad2/3^(−/−)^ MEF cells and Smad2/3^(−/+)^ MEF cells were kindly gifted by Dr. Xiao Yang (Chinese PLA Academy of Military Medical Sciences, Beijing, China) and were similarly cultured in Dulbecco’s modified Eagle’s medium with the exception of serum (15% fetal bovine serum). CKI-ε (WT and KD mutant) constructs were provided by Dr. Xiaofan Wang (Department of Pharmacology and Cancer Biology, Duke University Medical Center, Durham, North Carolina, USA). Patient tissue samples were provided by Dr. Junbo Hu at Tongji Hospital, Wuhan, China).

### Apoptosis assays

Apoptosis was examined by detecting phosphatidylserine (PS) exposure on cell membrane with Annexin V and dye exclusion assay as described previously (Chen et al., 2001). Cells were simultaneously stained with Annexin V-FITC (green) and PI (red). This assay discriminates between intact (FITC^−^/PI^−^), early apoptotic (FITC^+^/PI^−^), and later apoptotic cells (FITC^+^/PI^+^). Comparative experiments were performed at the same time by bivariate flow-cytometry using a FACScan (BD) and analyzed with CellQuest software on data obtained from the cell population from which debris was gated out. Nucleus condensation or DNA fragmentation was detected to indicate apoptosis in some experiments using DAPI/Hoechst staining. Briefly, cells were washed with PBS and stained with DAPI or directly stained with Hoechst 33342 before visualization under fluorescent microscopy. At least 200 cells from 6 random selected areas were counted in each experiment. Caspase-3 activation was determined by detecting its cleaved fragments using Western blotting. Pro-caspase-3 (37 kDa) was cleaved into 17 kDa fragment (cleaved caspase-3) during apoptosis.

### Co-immunoprecipitation (co-IP)

Two near confluent 75-mm dishes of Hep3B cells were washed three times with phosphate buffered saline (PBS), collected and lysed with 500 μL ice cold lysis buffer (50 mmol/L Hepes, pH 7.5, 150 mmol/L NaCl, 5 mmol/L EDTA and 1% Triton X-100) containing protease inhibitor cocktail (10 μg/mL Aprotinin, 1 nmol/L PMSF and 10 μg/mL Leupeptin) for 30 min at 4°C. Lysates were clarified by centrifugation at 15,000 ×*g* for 15 min and pre-cleared by incubation with protein G Plus-Sepharose for 120 min at 4°C. After pre-clearing, supernatants were transferred to 1.5-mL microfuge tubes containing anti-Smad2/3 mAb plus protein G-Sepharose. After incubation with rotating overnight at 4°C, immunoprecipitates were washed three times with RIPA lysis buffer and subjected to Western blotting analysis with anti-FoxO3 pAb and anti-Smad4 mAb.

### Western blotting analysis

Western blotting was performed according to our published method (Liao et al., 2003). Hep3B cells were harvested and lysed in lysis buffer (in mmol/L: 25 HEPES, pH 7.4, 5 EDTA, 8.0 EGTA, 1.0 Na_3_VO_4_, 0.25 NaF, 0.1 phenylmethylsulfonyl fluoride, 1.0 dithiothreitol; and 1% NP-40, 5 μg/mL aprotinin, 100 μg/mL leupeptin, 50 μg/mL trypsin inhibitor). Cellular protein (20 μg) was loaded and separated on sodium dodecyl sulfate polyacrylamide gel (BioRad mini gel, 6%–12% according to target protein molecular weight) and transferred to a nitrocellulose membrane (GibcoBRL) by the standard electric transfer protocol. The membrane was blocked at room temperature with PBS containing 0.1% Tween-20 (PBST) plus 5% non-fat milk for 120 min, probed with antibodies overnight at 4°C, then incubated with horseradish peroxidase-labeled second antibody (KPL Corp.) in blocking buffer for 120 min at room temperature. The membrane was then exposed to an enhanced chemiluminescent system and autoradiography was used to visualize immuno-reactive bands.

### Immunofluorescence double staining

Hep3B cells were plated onto 12-mm diameter round glass cover slips in six-well plate. Next day cells were incubated with TGF-β (5 ng/mL), then washed three times with PBS and fixed with 3.7% paraformaldehyde for 15 min at 37°C (As for mitochondria staining, Mito-Tracker was added into culture medium before fixation). Fixed cells were rinsed with PBS and incubated with 50 mmol/L NH_4_Cl for 10 min at 37°C. Cells were rinsed in PBS, sequentially permeabilized with 0.2% Triton X-100 on ice for 5 min, and washed with PBS for 5 min each at room temperature. After incubation for 60 min in blocking buffer, cells were incubated with primary antibodies for 60 min at room temperature or overnight at 4°C (primary antibody IgG was diluted into PBS with 0.05% Triton X-100 and 0.2% BSA). After three washes with PBS (5 min each), cells were incubated with the secondary antibodies for 60 min at room temperature: fluorescein isothiocyanate (FITC) conjugated goat anti-rabbit IgG (1:200) and Cy3 conjugated donkey anti-mouse IgG (1:200). Cells were washed three times with PBS (5 min for each wash). Cells on cover slips were mounted with slow-fade anti-fade reagent containing DAPI onto glass slides and were observed under fluorescent microscopy.

### Semiquantitative reverse transcription-polymerase chain reaction (RT-PCR)

To determine the mRNA levels of the concerned genes, we isolated total RNA from cells using Trizol (Invitrogen). Reverse transcription reactions were carried out with 5 μg of total RNA following the standard protocol supplied with the reverse transcriptase. The resulting cDNA was used for PCR and GAPDH was used as a loading control. The primers employed for each gene are listed below. All the reactions had a hot start of 5 min at 95°C and a final elongation step at 72°C for 10 min.

GAPDH: 5′-GGTATCGTGGAAGGACTCATGAC-3′ (sense) and 5′-ATGCCAGTGAGCTTCCCGTCAGC-3′ (antisense); Bim: 5′-ATGGCAAAGCAACCTTCTGA-3′ (sense) and 5′-TCAATGCATTCTCCACACCA-3′ (antisense); Bcl-xL: 5′-ATGTCTCAGAGCAACCGGGAGC-3′ (sense) and 5′-TTTCCGACTGAAGAGTGAGCCCA-3′ (antisense); Bax: 5′-ACCAAGAAGCTGAGCGAGTGTC-3′ (sense) and 5′-ACAAAGATGGTCACGGTCTGCC-3′ (antisense).

### SiRNA interfering

SiRNA transfection was performed according to the manufacturer’s instructions. Briefly, cells were plated at a density of 5 × 10^5^ cells/well in 6-well plates. Cells were transfected with 80 nmol/L siRNA duplex mixture (Cell Signaling Biotechnology, Beverly, MA) for 24 h in the presence of lipofectamine RNAiMax (Invitrogen Inc., Carlsbad, CA). A nonspecific control siRNA (Scramble siRNA) (Cell Signaling Biotechnology, Beverly, MA) was also transfected at the same concentration as the negative control.

### Statistical analysis

All experiments were performed at least 3 times, and results are reported as mean ± 95% confidence intervals unless otherwise stated. A *P* < 0.05 was considered statistically significant.

## ABBREVIATIONS

Bax: Bcl2-associated X protein
Bcl-xL: B-cell lymphoma-xL
Bim: Bcl2-interacting mediator of cell death
CKI-ε: casein kinase I-ε
DAPI: 4,6-Diamidino-2-phenyindole
FITC: fluorescein isothiocyanate
FoxO: Forkhead box protein class-O
IGF-1: Insulin-like growth factor-1
mAb: monoclonal antibody
pAb: polyclonal antibody
PBS: phosphate buffered saline
PI: Propidium Iodide
PI3K: phosphoinositide 3-kinase
siRNA: small interfering RNA
Smad: the *C. elegans* protein ‘SMA’ and mothers against decapentaplegic ‘MAD’
TGF-β: transforming growth factor-β
